# Poly(glycerol)-Functionalized Gadolinium Tungstate Nanoflakes Loaded with Chlorin e6: Photodynamic Efficacy and Radiosensitization Potential for Multimodal Cancer Therapy

**DOI:** 10.3390/ma18225198

**Published:** 2025-11-16

**Authors:** Heon Gyu Kang, Lukas R. H. Gerken

**Affiliations:** 1 Graduate School of Human and Environmental Studies, Kyoto University, Sakyo-ku, Kyoto 606-8501, Japan; 2 Particles-Biology Interactions, Department Materials Meet Life, Swiss Federal Laboratories for Materials Science and Technology (Empa), Lerchenfeldstrasse 5, 9014 St. Gallen, Switzerland; gerken.lrh@gmail.com

**Keywords:** gadolinium tungstate, biomedicine applications, radiotherapy, photodynamic therapy

## Abstract

Gadolinium (Gd)-based nanomaterials have attracted a considerable amount of attention in cancer treatment research due to their applicability in radiotherapy. However, the clinical translation of Gd-based nanomaterials is limited by their high density and poor dispersibility in aqueous media, thereby necessitating surface functionalization with biocompatible polymers. In this study, gadolinium tungstate (Gd_2_(WO_4_)_3_) nanoflakes (GW Nfs) were functionalized with poly(glycerol) (PG) to enhance their dispersibility and stability in aqueous media. Due to their high-*Z* elemental composition, the GW Nfs generated reactive oxygen species (ROS) under X-ray irradiation, with improved dispersibility induced by PG functionalization further enhancing ROS productivity compared to GW Nfs. Furthermore, PG-GW loaded with the photosensitizer chlorin e6 (Ce6) demonstrated strong photocytotoxicity at Ce6 concentrations as low as 0.2 μg mL^−1^ under light irradiation. Taken together, these results demonstrate that PG-GW/Ce6 is a promising nanomaterial for photodynamic therapy while also offering prospects for bimodal photon cancer therapy with X-rays.

## 1. Introduction

Gadolinium (Gd)-based materials have attracted a considerable amount of attention in the field of nanomedicine for cancer, as the unique physicochemical properties of Gd enable multiple modalities [1,2]. In particular, its high atomic number (*Z* = 64) confers strong X-ray absorption, facilitating both computed tomography (CT) imaging and radiotherapy [3,4]. Moreover, the isotope ^157^Gd exhibits an exceptionally large thermal neutron capture cross section, which has been exploited in gadolinium neutron capture therapy (Gd-NCT) [1,5,6]. Despite their potential for use in cancer diagnosis and therapy, Gd-based nanomaterials often suffer from limited aqueous dispersibility, restricting their direct use in physiological environments [7,8]. Surface modifications of Gd_2_(WO_4_)_3_-based nanoparticles, such as SiO_2_ and poly(ethylene glycol) (PEG) coatings, have been explored [9] but typically without a detailed evaluation of colloidal stability. In contrast, poly(glycerol) (PG) forms a hydroxy-rich, highly hydrophilic shell that improves water dispersibility and can minimize protein adsorption, as shown in previous studies [10], and it has also been used to modify various nanoparticles for biomedical applications [11,12,13,14,15]. In particular, ^10^B-enriched nanoparticles with PG, such as ^10^B_4_C-PG and h-^10^BN-PG, exhibited high tumor accumulation and enhanced boron neutron capture therapy (BNCT) efficacy [11,12].

In this study, gadolinium tungstate (Gd_2_(WO_4_)_3_) nanoflakes (GW Nfs) were functionalized with PG to enhance aqueous dispersibility [16]. The high hydrophilicity and multibranched structure of PG rendered the PG-functionalized GW Nfs (PG-GW) stably dispersible in physiological media, paving the way for biomedical applications such as radiotherapy. Notably, PG-GW loaded with chlorin e6 (Ce6) maintained high aqueous dispersibility and exhibited pronounced phototoxicity even at low Ce6 concentrations, demonstrating its potential as a drug carrier for photodynamic therapy (PDT) as well as a sensitizer for X-ray radiotherapy.

## 2. Experimental Section

### 2.1. Reagents

Gadolinium (III) nitrate [Gd(NO_3_)_3_], Sodium tungstate dihydrate (Na_2_WO_4_·2H_2_O), urea, chlorin e6 (Ce6), and glycidol were obtained from Sigma-Aldrich Chemie GmbH (Buchs, Switzerland).

### 2.2. Characterizations

SEM was performed using an Axia ChemiSEM (Thermo Fisher Scientific, Waltham, MA, USA) operated at 2 kV. TEM imaging was performed using a Zeiss EM 900 microscope (Carl Zeiss Microscopy GmbH, Jena, Germany) operated at 80 kV. AFM analysis was conducted using a Bruker Dimension Icon AFM (Billerica, MA, USA). FTIR spectra were recorded on a Vertex 80v spectrometer (Bruker Optics Inc., Billerica, MA, USA). Weight loss was monitored in the temperature range of 25–1000 °C via TGA in thermobalance (TGA/SDTA851e, LF/1100 °C, Mettler Toledo AG, Greifensee, Switzerland). XRD analysis was performed with a Bruker D2 2nd Gen Phaser (30 kV, 10 mA, SSD160 detector, Cu tube 1.54184 Å Kα radiation at 2*θ* = 10°−80° with a step size of 0.01°, Bruker Corporation, Billerica, MA, USA). Phase composition and crystallite sizes were determined using Diffrac Eva (V3.1) software after performing Rietveld parameter refinement. Hydrodynamic size of PG-GW (dynamic light scattering, DLS) was measured using a Zetasizer Nano ZS90 (Malvern Instruments Ltd., Worcestershire, UK) in number mode. PDI was calculated as (SD/mean)^2^ [17]. UV–vis–NIR absorption spectra were recorded on a UV-3100PC scanning spectrophotometer (Shimadzu Co., Ltd., Kyoto, Japan). PG-GW/Ce6 and PG-GW (centrifuged at 1000× *g*) DLS analysis were conducted five times for each sample using a Nanotrac UPA-UT151 system (MicrotracBEL GmbH, Haan, Germany) in number mode, and PDI was calculated as (SD/mean)^2^ [17]. Zeta potential measurements were performed five times by using a Zetasizer Nano ZS90 (Malvern Instruments Ltd., Worcestershire, UK). Cell viability assays were performed using an MTP-310 microplate reader (Corona Electric Co., Ltd., Ibaraki, Japan) equipped with a 450 nm wavelength filter.

### 2.3. Preparation Methods of BM-GW Nfs

Gadolinium tungstate nanoflakes (GW Nfs) were prepared using a coprecipitation method, as described in a previous report [18]. Initially, 0.1 M Gd(NO_3_)_3_ and 0.2 M Na_2_WO_4_ were dissolved in 200 mL of DI water. With constant stirring at 750 rpm, the morphological influence was improved by adding 0.5 M urea (0.6 g in 20 mL) to the above solution and continuously stirring it for 1 h at room temperature to obtain a white precipitate. Subsequently, the product obtained was washed with ethanol and DI water by using a centrifuge. After thorough washing, the precipitate was dried in vacuum oven at 50 °C for 18 h. Finally, the collected samples were annealed in an air atmosphere at 800 °C for 4 h (heating rate of 5 °C min^−1^). To restore surface hydroxy groups, GW Nfs were wet ball-milled in water (ball-to-powder ratio 10:1) at 800 rpm for 8 h by using Pulverisette 7 (Fritsch, Idar-Oberstein, Germany). The resulting dispersion was centrifuged at 78,000× *g*, the supernatant was discarded, and the pellet was dried in a vacuum oven overnight.

### 2.4. Synthesis of PG-GW

BM-GW Nfs powder (10 mg) was dispersed in glycidol (20 mL) via bath sonication for 1 h and then heated at 140 °C with magnetic stirring for 20 h. After cooling down, the yellow gel was redispersed in 40 mL of water via bath sonication. The crude product was collected via centrifugation (78,000× *g*, 1 h) and then purified in water via a repeated redispersion/ultracentrifugation cycle to remove the detached free poly(glycerol) in the supernatant. The PG-GW obtained was redispersed in water.

### 2.5. ROS Assay

To assess the quantity of ROS, mostly hydroxyl radicals, generated during X-ray irradiation, the DCF assay was performed according to the method reported in reference [19]. In short, a 5 mM stock solution was prepared from 2′,7′-Dichlorodihydrofluorecein diacetate powder (H_2_DCF–DA, Sigma-Aldrich) and dimethyl sulfoxide (DMSO, Sigma-Aldrich). The H_2_DCF-DA stock solution was incubated with 4 parts of 10 mM NaOH for 30 min in the dark at room temperature before it was diluted 1:100 with PBS buffer, subsequently reaching a final 10 μM fluorophore concentration and kept protected from light. Dry nanomaterial powders were weighed and sonicated in Milli-Q water. Immediately prior to irradiation with 0 or 13 Gy (150 kVp X-rays, 1.5 Gy dose rate, Seifert ISOVOLT 450, GE Sensing & Inspection Technologies GmbH, Hürth, Germany), 1 part of 10 µM H_2_DCF working solution was added to 1 part of nanomaterial-suspension. After irradiation, all tubes were centrifuged (21,000× *g*, 10 min, 14 °C), and 40 µL supernatants were transferred in triplicate into a 96-well plate and protected from light until fluorescence was measured using a microplate reader (485/535 nm excitation/emission, Mithras LB 943 Multimode Reader, Berthold Technologies, Bad Wildbad, Germany). The ROS enhancement factors were calculated by subtracting the 0 Gy from the 13 Gy Fluorescence signal and dividing the subtracted values pertaining to nanomaterial conditions by those concerning nanomaterial-free conditions.

### 2.6. Physical Dose Enhancement Calculations

The macroscopic physical dose enhancement factor (DEF) was approximated using calculations provided by reference [20]:

$${\mathrm{DEF}}_{NP}=1+f_{NP}\frac{\int_{E=0}^{E_{max}}\Phi^{\prime }\left(E\right)\;E{\;\left(\frac{\mu_{en}\left(E\right)}{\rho }\right)}_{NP}dE}{\int_{E=0}^{E_{max}}\Phi^{\prime }\left(E\right)\;{E\;\left(\frac{\mu_{en}\left(E\right)}{\rho \;}\right)}_{H_{2}O}dE},$$

where *f*_NP_ denotes the mass fraction of the nanomaterial (here: Gd_2_(WO_4_)_3_) in the nanomaterial–water mix and *µ*_en_(*E*)/*ρ* denotes the mass energy-absorption coefficient at X-ray energy E obtained from the NIST Standard Reference Database [21]. The polyenergetic photon spectrum for the 150 kVp X-ray tube used and its differential photon fluence $\Phi^{\prime }\left(E\right)$ are provided in reference [22]. The energies were discretized and sampled in the 0–150 keV range at increments of approximately 1.1 keV. The mass energy absorption coefficients at each of these energies were calculated via linear interpolation between the data points provided by NIST. The elemental mass fractions in the Gd_2_(WO_4_)_3_ material were assumed to be approximately 29.7% Gd, 52.1% W, and 18.1% O. MATLAB_R2024b (MathWorks Inc., Natick, MA, USA) was used for the calculations.

### 2.7. Loading Ce6 onto PG-GW

The PG-GW (1 mL of 1.2 mg mL^−1^) and Ce6 (2.4 mg) in Milli Q water (2 mL) were ultrasonicated using a PR-1 nanoparticle dispersion system (Thinky Corporation, Tokyo, Japan) at 160 mW, 15 °C, and 100 rpm rotation for 3 h, following a previously reported method [23,24]. After sonication, the dispersion was centrifuged at 1000× *g* for 1 h, and the supernatant was collected. The supernatant was further centrifuged under the same conditions three times to remove free Ce6.

### 2.8. The Loading Capacity of Ce6

The absorbance of the PG-GW/Ce6 dispersion (Figure 5b in the main text) consists of baseline absorbance of PG-GW (blue-dashed-line curve) and absorbance of Ce6. The loading capacity of Ce6 was determined using the Beer-Lambert law:

$$A=ɑ\times C_{Ce6}\times l$$

where *A*, *ɑ*, *C*_Ce6_, and *l* are the absorbance (at Q(I) band, 655 nm), absorption coefficient, Ce6 concentration (mg mL^−1^), and cell length (1 cm), respectively. The linear relationship between *A*/*l* and *C* yields an absorption coefficient (*ɑ*_Ce6_) of 4910 mL mg^−1^ m^−1^ at the Q(I) band, as previously reported [23]. The concentration of PG-GW was derived by subtracting *C*_PG-GW/Ce6_ from the dry weight of PG-GW/Ce6 and the substrate *C*_Ce6_. Our estimate of the *ɑ*_Ce6_ of free Ce6, determined above, was quite similar to that of Ce6 in the PG-GW/Ce6.

$$A_{Ce6}=n_{diluted\;times}\times (A_{PG-GW/Ce6}-A_{PG-GW})=20\times (0.04305-0.00303)=0.8004$$

$$C_{Ce6}=A_{Ce6}\;/\;a_{Ce6}\;l=0.0163\;mg\;{mL}^{-1}$$

$$C_{PG-GW}=C_{PG-GW/Ce6}-C_{Ce6}=\left(0.90-0.0163\right)\;mg\;{mL}^{-1}=0.88\;mg\;{mL}^{-1}$$

The loading capacity of Ce6 (*C*_Ce6_/*C*_PG-GW_) was determined to be 1.9 wt%.

### 2.9. Decomposition Rate Constant Calculation

The decomposition rate constant of photosensitizer (*k*_PS_) was determined using a pseudo-first-order reaction model, based on the linear regression of ABDA absorbance decay at 400 nm over time during 660 or 690 nm laser irradiation, using free Ce6 (15 µg mL^−1^) and PG-GW/Ce6 (8 µg mL^−1^ Ce6 equivalent). The pseudo-first-order reaction is expressed as

$$k_{PS}=-ln\left(\frac{A_{t}}{A_{0}}\right)\times t^{-1}$$

where *A_t_* and *A*_0_ are the absorbance values of ABDA at *t* and time *t* = 0, respectively, and *k*_PS_ is the pseudo-first-order decomposition rate constant. Based on the slopes of the linear plots shown in Figure S6, the *k*_Ce6+690_, *k*_PG-GW/Ce6+660_, and *k*_PG-GW/Ce6+690_ were determined to be 5.9 × 10^−4^ s^−1^, 7.7 × 10^−4^ s^−1^, and 1.2 × 10^−3^ s^−1^, respectively.

### 2.10. Phototoxicity of PG-GW/Ce6

CT26 and 4T1 cells were seeded in 96-well plates with a density of 1 × 10^4^ cells per well and incubated for 24 h. The cells were treated with PG-GW/Ce6 at Ce6 equivalent concentrations of 0.1, 0.2, and 0.4 µg mL^−1^. After incubation for 24 h, two irradiation conditions were compared: (i) cells irradiated immediately without washing, followed by 15 min incubation and subsequent replacement with fresh culture medium, and (ii) cells washed twice with PBS (100 μL) and replenished with fresh culture medium (100 μL) prior to irradiation. For determining phototoxicity, cells were irradiated in each well using a fiber-coupled laser (Changchun New Industries Optoelectronics Technology Co., Ltd., Changchun, China) with wavelengths of 660 nm and 690 nm (1.3 W cm^−2^ for 30 s). Cell viability, with or without light exposure, was measured 24 h post-incubation with a CCK-8 kit, as instructed in the manual provided by the kit’s manufacturer (Dojindo Molecular Technologies, Inc., Kumamoto, Japan). CT26 and 4T1 cells between passages 6 and 9 were used in all experiments. For live/dead cell imaging, CT26 cells were seeded in 12-well plates with a density of 1 × 10^4^ cells per well and incubated for 24 h. The cells were treated with PG-GW/Ce6 at Ce6 equivalent concentrations of 0.12 µg mL^−1^. After incubation for 24 h, cells were washed twice with PBS (1 mL) and replenished with fresh culture medium (1 mL) prior to irradiation. The cells were irradiated in each well using a fiber-coupled laser (Changchun New Industries Optoelectronics Tech. Co., Ltd., Changchun, China) with a wavelength of 660 nm (1.3 W cm^−2^ for 30 s). After incubation for 24 h, cells were stained with 0.04% trypan blue in PBS (Fujifilm Co., Tokyo, Japan) instead of CCK-8, and images were acquired via optical microscopy at 10× magnification. CT26 cells between passages 11 and 12 were used in all experiments.

## 3. Results and Discussion

**Figure 1:**
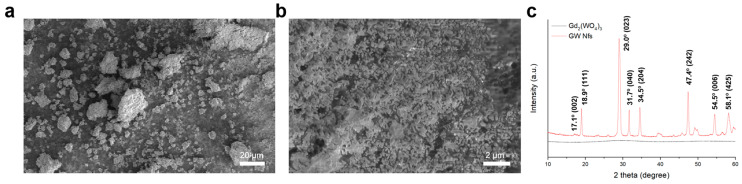
(**a**) SEM image of Gd_2_(WO_4_)_3_ (scale bar: 20 μm), (**b**) SEM image of annealed GW Nfs (scale bar: 2 μm), and (**c**) XRD patterns of Gd_2_(WO_4_)_3_ and GW Nfs.

**Figure 2:**
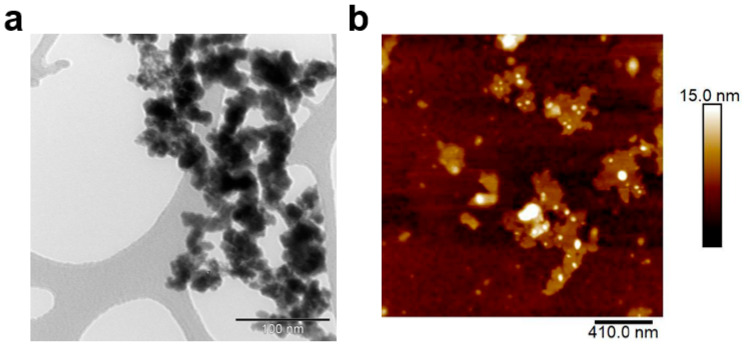
(**a**) TEM image (scale bar: 100 nm) and (**b**) AFM image (scale bar: 410 nm) of GW Nfs.

Gd_2_(WO_4_)_3_ and GW Nfs were prepared using a coprecipitation and annealing method, as previously reported (details are given in Section 2) [18]. Scanning electron microscope (SEM) images showed that Gd_2_(WO_4_)_3_ appeared as dense aggregates, whereas the GW Nfs exhibited a flake-like morphology (Figure 1a,b). Energy-Dispersive X-Ray Spectroscopy (EDS) analysis was performed on the SEM images of the GW Nfs (Figure S1). The atomic concentrations of Gd and W were 4.36% and 6.96%, respectively, consistent with the 2:3 stoichiometry of Gd_2_(WO_4_)_3_. X-ray diffraction (XRD) patterns of the Gd_2_(WO_4_)_3_ showed no distinct diffraction peaks, indicating its non-crystalline nature. In contrast, the GW Nfs displayed sharp diffraction peaks corresponding to the monoclinic crystal structure of Gd_2_(WO_4_)_3_, which is consistent with previously reported GW Nfs [18]. Transmission electron microscopy (TEM) and atomic force microscopy (AFM) further verified the nanoflake morphology, with lateral dimensions of several hundred nanometers and a thickness of up to 15 nm, corresponding to a two-dimensional (2D) structure (Figure 2). These observations confirm the successful preparation of nanoflakes with structural characteristics comparable to those of the previously reported GW Nfs [18].

**Figure 3:**
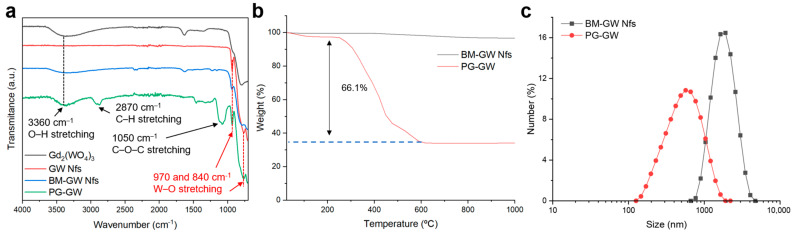
(**a**) FTIR spectra of bulk Gd_2_(WO_4_)_3_ (black line), GW Nfs (red line), BM-GW Nfs (blue line), and PG-GW (green line). (**b**) TGA profile of BM-GW Nfs (black) and PG-GW (red) measured in a nitrogen atmosphere. (**c**) DLS profiles (number mode) of BM-GW Nfs and PG-GW dispersed in Milli-Q water.

Fourier transform infrared (FTIR) analysis showed that the characteristic –OH peak at 3500 cm^−1^ in Gd_2_(WO_4_)_3_ disappeared in the GW Nfs after annealing (Figure 3a), suggesting that the ring-opening polymerization of glycidol cannot be initiated for PG functionalization [14,25]. To overcome this limitation, ball milling was carried out in water. As shown in Figure 3a, the characteristic –OH absorption band at 3500 cm^−1^ reappeared in the ball-milled (BM)-GW Nfs, indicating regeneration of surface hydroxy groups. Compared to pristine GW Nfs, the BM-GW Nfs exhibited decreased intensity and broadening of the XRD peaks, indicative of reduced crystallinity (Figure S2a). However, the diffraction pattern confirmed that the monoclinic Gd_2_(WO_4_)_3_ phase was preserved, as in previous reports on wet ball-milled zeolites [26]. AFM analysis showed that the lateral size and thickness, or nanoflake morphology, of BM-GW Nfs are not so different from those of GW Nfs (Figure S2b). After PG coating, the characteristic bands of PG-GW at 2900 and 1100 cm^−1^, corresponding to C–H and C–O–C stretching vibrations, appeared, as shown in Figure 3a, confirming the successful introduction of PG [11,12,15]. Thermogravimetric analysis (TGA) revealed 66% weight loss in the range of 250–600 °C (Figure 3b), further supporting successful PG functionalization. Dynamic light-scattering (DLS) measurements revealed that the BM-GW Nfs exhibited an average hydrodynamic size of 1884.8 ± 628.7 nm with a polydispersity index (PDI) of 0.11 (Figure 3c). The BM-GW Nf particle size was estimated to be ~400 nm based on the AFM images (Figure S2b), so the larger hydrodynamic size may originate from aggregation of BM-GW Nfs in water. The BM-GW Nfs at 1.2 mg mL^−1^ formed precipitates in water within 2 h, whereas PG-GW was well-dispersed without precipitation (Figure S3). PG-GW showed a smaller average hydrodynamic size of 568.3 ± 299.8 nm with a PDI of 0.28 and good dispersibility in water at concentrations as high as 1.2 mg mL^−1^ without precipitation for up to one year. These findings demonstrate that PG functionalization markedly enhances the colloidal stability of BM-GW Nfs in aqueous solutions despite the relatively large particle size and density.

**Figure 4:**
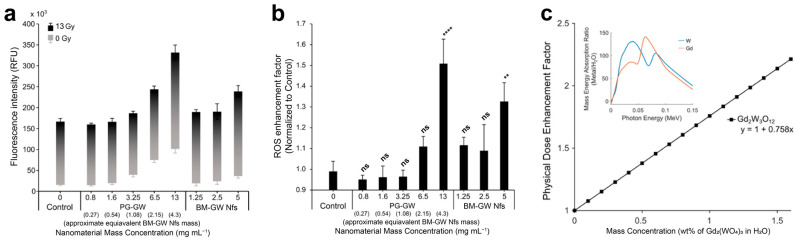
X-ray (150 kilovolt peak, kVp) sensitizability of BM-GW Nfs and PG-GW: (**a**) Fluorescence intensity (RFU: relative fluorescence unit) of DCF in H_2_O/PBS solutions with or without BM-GW Nfs or PG-GW after 0 and 13 Gy X-ray irradiation treatment. (**b**) ROS enhancement of BM-GW Nfs and PG-GW relative to control solutions. Statistical analysis was performed using one-way ANOVA followed by Tukey’s post hoc test. Asterisks and ns indicate significance levels in comparison to the control group (** *p* < 0.01; **** *p* < 0.0001; ns = not significant). (**c**) Physical (macroscopic) dose enhancement factor per Gd_2_(WO_4_)_3_ mass concentration for a 150 kVp X-ray source theoretically calculated from the mass energy absorption ratios of Gd, W, and O compared to H_2_O. Error bars represent the mean ± SD of three technical replicates.

Given the presence of two high-*Z* elements (Gd and W), the radiosensitizing potential of BM-GW Nfs and PG-GW was evaluated through X-ray irradiation experiments and dose enhancement calculations. The high photon interaction cross section of the BM-GW Nfs and PG-GW with kilovoltage X-rays (kV X-rays) leads to secondary electron emission, especially of photo- and Auger electrons [27]. These electrons facilitate the generation of reactive oxygen species (ROS), especially hydroxyl radicals, from the water molecules within ~200 nm of the nanomaterials. ROS generation efficiency during X-ray irradiation was assessed using the 2’,7’-dichlorofluorescein (DCF) assay [28]. Irradiation without materials led to an increase in ROS levels as indicated by the increase in DCF fluorescence intensity after 13 Gy X-ray treatment (control in Figure 4a). Compared to the control, the BM-GW Nfs and PG-GW led to a material dose-dependent increase in DCF fluorescence, or ROS generation, at a concentration of ≥1.25 mg mL^−1^ of BM-GW Nfs (Figure 4b) [19,29]. However, statistical analysis (one-way ANOVA followed by Tukey’s post hoc test) revealed that only the highest concentrations of PG-GW (13 mg mL^−1^) and BM-GW Nfs (5 mg mL^−1^) showed statistically significant enhancement over the control. While the low-dose groups did not reach statistical significance, the overall trend is suggestive of a dose-dependent response. Therefore, linear regression was applied to explore the strength of the correlation (Figure S4), with the understanding that this does not imply significance at each point. Although a higher ROS signal for PG-GW compared to that for BM-GW Nfs was observed at the highest concentration, 13 mg mL^−1^, the regression trend nevertheless indicated that PG-GW exhibited greater ROS enhancement than BM-GW Nfs (Figure S4). This is likely due to the difference in hydrodynamic sizes between BM-GW Nfs and PG-GW [30]. The hydrodynamic diameter decreased from 1884.8 nm (BM-GW Nfs) to 568.3 nm (PG-GW), while the mass-normalized ROS-enhancement slope increased from 0.609 to 0.953. Compared to the theoretical DEF of 0.758 (Figure 4c, detailed in Section 2), the BM-GW Nfs showed a slightly lower level, while that for PG-GW was slightly higher. This inverse trend suggests that aggregation in BM-GW Nfs limited dose enhancement, whereas PG-GW indicated smaller size and better dispersibility, enabling greater ROS generation per mass. However, homogeneous dispersion may not fully explain the value exceeding the theoretical DEF. A likely additional factor is the larger specific surface area of PG-GW, which could enhance ROS generation beyond DEF predictions. In comparison, TiO_2_ or WO_3_ nanoparticles [19,22] can yield ROS enhancements 10–100 times higher than the theoretical DEF through surface charge transfer catalysis. By contrast, the ROS enhancement of PG-GW showed only a 1.2-fold increase, indicating that secondary-electron mediation is the dominant mechanism. To assess radiosensitizing performance, the ROS enhancement factor of PG-GW was compared with that of previously reported Gd-based nanoparticles. Activation and Guidance of Irradiation by X-rays (AGuIX) showed enhancement factors ranging from 1.1 to 2.5, and Gd_2_O_3_@SiO_2_ exhibited a value of approximately 1.83 at a Gd concentration of 48 µg mL^−1^ [31,32]. At the same Gd concentration in the PG-GW system (0.16 wt%), the ROS enhancement factor was estimated to be 1.2 based on the regression trend in Figure S4. This factor is the lower range of the above values reported for AGuIX. While this does not reflect a statistically validated enhancement at that specific concentration, it suggests that PG-GW may offer comparable radiosensitization potential at similar Gd concentrations.

**Figure 5:**
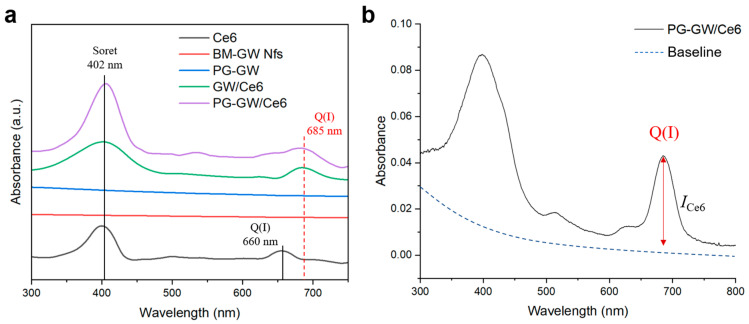
(**a**) UV–vis absorption spectra of Ce6 (black), BM-GW Nfs (red), PG-GW (blue), GW/Ce6 (green), and PG-GW/Ce6 (purple) in milli-q water. (**b**) Absorption spectrum of PG-GW/Ce6 in milli-q water with the baseline indicated by a dashed blue line.

PG-GW was further evaluated as a drug carrier by loading the PDT photosensitizer chlorin e6 (Ce6) via sonication to obtain PG-GW/Ce6 [23]. For comparison, BM-GW Nfs were also loaded with Ce6 under the same conditions, and the resulting GW/Ce6 was analyzed using UV–vis spectroscopy (Figure 5a). While free Ce6 exhibited a Q-band at around 660 nm, both GW/Ce6 and PG-GW/Ce6 showed a red-shifted Q-band at 685 nm. A similar red shift has been reported for graphene/Ce6 composites, suggesting that this phenomenon may originate from interactions between Ce6 and the BM-GW Nfs rather than PG [23]. BM-GW Nfs lack extended conjugated π-systems, and thus π–π interaction is unlikely, although it is also a form of van der Waals interaction. Instead, the observed Q-band red shift is presumed to originate from other types of van der Waals forces, such as dipole-induced dipole interaction (Debye force) and London dispersion interactions [33], occurring between Ce6 and the BM-GW surface. Supporting this, a similar Q-band red shift was also reported for tetra(4-carboxyphenyl)porphyrin (TCPP), which contains a porphyrin ring structure similar to that of Ce6, adsorbed on hexagonal boron nitride (h-BN). Theoretical calculations identified van der Waals interactions as the primary binding mechanism [34,35]. The Ce6 loading content of PG-GW/Ce6 was determined to be 16 μg mL^−1^ based on the absorption spectra (Figure 5b); this result was obtained by following a previously reported method [23]. Accordingly, the Ce6 contents were determined to be 1.9 and 5.6 wt% relative to the PG-GW and BM-GW Nf weights in PG-GW/Ce6. The relatively low loading, compared with that for graphene/Ce6 [23], may reflect (i) the much higher *Z* of the GW Nfs relative to graphene and (ii) Ce6–GW Nf contact hindered by PG in contrast to direct π–π interaction between Ce6 and graphene [36,37].

**Figure 6:**
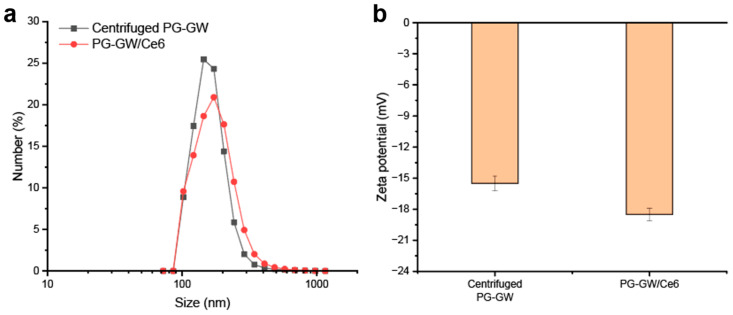
(**a**) DLS profiles of PG-GW after centrifugation at 1000× *g* for 1 h (black) and after Ce6 loading (red). (**b**) Corresponding zeta potentials. Error bars represent the mean ± SD of 5 technical replicates.

To ensure comparability, PG-GW/Ce6 and PG-GW were processed using the same centrifugation protocol (1000× *g*, 1 h) prior to all subsequent characterizations, including DLS (Figure 6a). This process is required for both materials because unbound Ce6 has to be washed out in PG-GW/Ce6. The average hydrodynamic size of PG-GW was 152.4 ± 37.7 nm with a PDI of 0.061, while that of PG-GW/Ce6 was 166.7 ± 51.0 nm with a PDI of 0.094, indicating that Ce6 loading does not induce aggregation. Meanwhile, zeta potential measurements revealed a shift from −15.5 ± 0.7 mV for PG-GW to −18.5 ± 0.6 mV for PG-GW/Ce6 (Figure 6b), reflecting a slightly more negative surface charge upon Ce6 loading. In previous studies, a Ce6 loading of about 80 wt% on GO led to a pronounced shift in the zeta potential from −40 to −50 mV [38]. Ce6 introduces carboxy groups on the particle surface, which partially dissociate to COO^–^ in an aqueous solution, making the zeta potential more negative. In contrast, PG-GW/Ce6 showed much less of a decrease, which can be attributed to the lower Ce6 loading capacity of 1.9 wt%, resulting in a much smaller contribution of carboxy groups to the overall surface charge relative to GO/Ce6.

**Figure 7:**
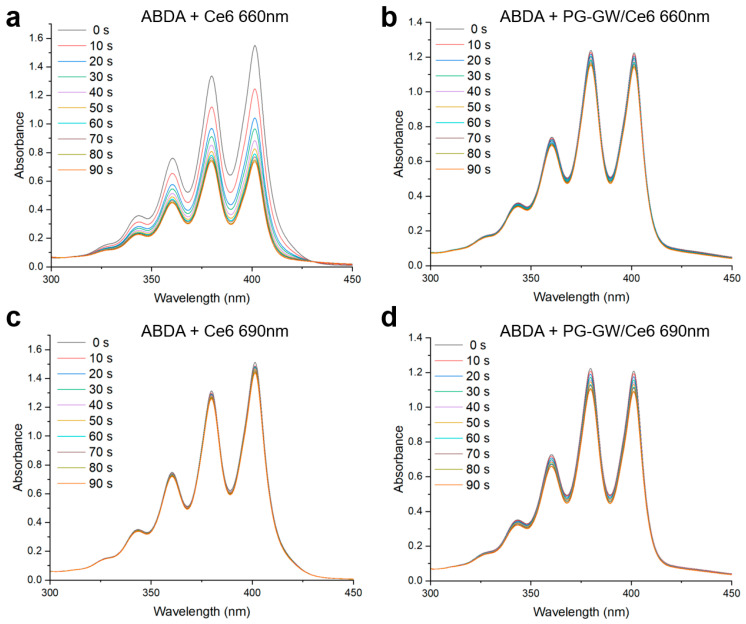
Time-resolved UV–vis spectra of ABDA in the presence of free Ce6 and PG-GW/Ce6 under continuous irradiation by a 660 nm (**a**,**b**) or 690 nm (**c**,**d**) laser (1.3 W cm^−2^). Spectra were recorded every 10 s from 0 to 90 s. The curves are color-coded according to irradiation time, corresponding to the labels shown in the legend.

Singlet oxygen production ability was evaluated using 9,10-anthracenediyl-bis(methylene)dimalonic acid (ABDA). The photon wavelengths at 660 and 690 nm were chosen to match the Q-bands of free Ce6 and the PG-GW/Ce6, respectively, as shown in Figure 5a. The Ce6 concentrations were 15 µg mL^−1^ for the ABDA + free Ce6 solution and 8 µg mL^−1^ in the ABDA + PG-GW/Ce6 solution (Figure 7 and Figure S5). The time-dependent decreases in the absorbance of ABDA at 400 nm were used to obtain pseudo-first-order decomposition rate constants (*k*_PS_) (Figure S6—the detailed calculations are in Section 2). While ABDA + Ce6 at 660 nm (Figure 7a) did not correspond to pseudo-first-order kinetics, probably due to much higher singlet oxygen productivity, *k*_PS_ was determined to be 5.9 × 10^−4^, 7.7 × 10^−4^, and 1.2 × 10^−3^ s^−1^ for ABDA + Ce6 at 690 nm, ABDA + PG-GW/Ce6 at 660 nm, and ABDA + PG-GW/Ce6 at 690 nm, respectively (Figure 7b–d and Figure S6). These results indicate that PG-GW/Ce6 exhibited higher singlet oxygen generation ability at 690 nm than at 660 nm, which is consistent with the red-shift of the Q band in the Ce6 through complexation with PG-GW (Figure 5). Moreover, the *k*_PS_ of ABDA + PG-GW/Ce6 at 660 nm is larger than that of ABDA + Ce6 at 690 nm, which can be attributed to the stronger absorption of PG-GW/Ce6 at 660 nm than Ce6 at 690 nm (Figure S5).

**Figure 8:**
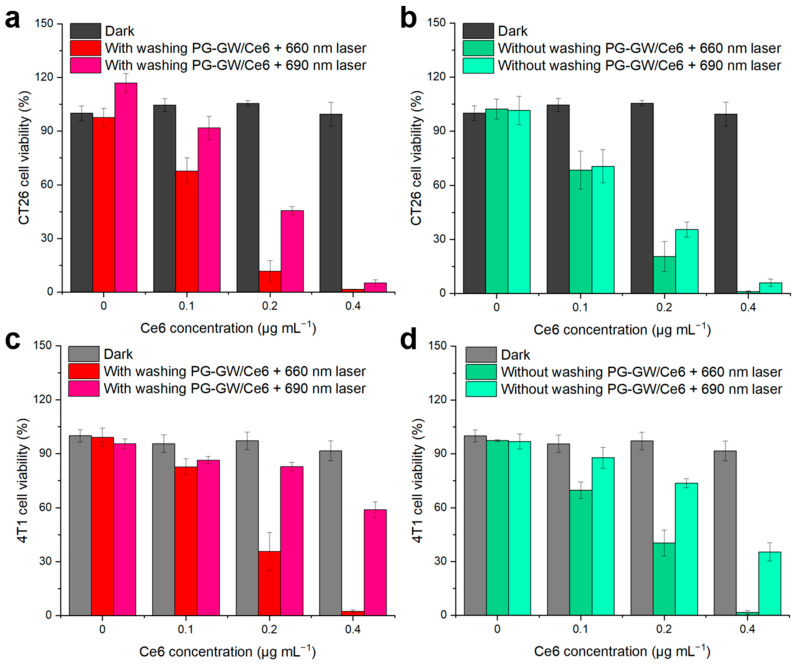
Cell viability after treatment with PG-GW/Ce6 at different concentrations: (**a**) CT26 cells that (**a**) were or were not (**b**) washed and 4T1 cells that (**c**) were or (**d**) were not washed before photon exposure. Cells were irradiated with 660 or 690 nm laser light (1.3 W cm^−2^, 30 s), and viability was determined using the CCK-8 assay. Error bars represent the mean ± SD of four parallel measurements.

The PDT efficacy of PG-GW/Ce6 was assessed in CT26 murine colorectal cancer cells (see Figure 8a,b, and the detailed procedure is given in Section 2) [12,39,40,41]. While PG-GW/Ce6 exhibited almost no toxicity toward CT26 cells in the dark, 88–98% and 55–95% of cancer cells were killed under 660 and 690 nm light irradiation, respectively, at Ce6 concentrations of ≥0.2 µg mL^−1^. In the groups of cells washed with PBS prior to laser irradiation (Figure 8a), the 660 nm laser induced higher photocytotoxicity than the 690 nm laser. This effect can be attributed to the intracellular uptake of PG-GW/Ce6, followed by the release of Ce6 under the acidic conditions in the lysosome (pH 4.5–5.0), as reported previously [13,42,43]. Ce6 exhibits strong Q band absorption at around 660 nm, whereas its absorption at 690 nm is weaker (Figure 5a). Therefore, 660 nm irradiation excites Ce6 more efficiently, generating ROS more efficiently and leading to higher photocytotoxicity (Figure 8a), although longer-wavelength light penetrates more deeply in vivo. To substantiate this interpretation, the dose–response relationship was quantified in the washed PG-GW/Ce6 under 660 nm laser irradiation (Figure 8a) and corroborated by live/dead dell imaging. The 50% inhibitory concentration (IC_50_) for PDT was calculated to be 0.12 µg mL^−1^ (Figure S7). Live/dead cell images of cells treated at this concentration are shown in Figure S8. After trypan blue staining, the controls without material or light (Figure S8a), and with 660 nm light irradiation alone (Figure S8b) and PG-GW/Ce6 addition alone (Figure S8c), were mostly transparent, indicating live cells. In contrast, the combined treatment with PG-GW/Ce6 addition and 660 nm light irradiation (Figure S8d) resulted in approximately half of the cells showing blue coloration, indicating cell death. These images confirm that treatment at the IC_50_ for PDT induces partial cell death, consistent with the quantitative cytotoxicity shown in Figure 8a. When the cells were not washed before irradiation, the 660 and 690 nm laser groups showed cytotoxicity (79–98% in Figure 8b), which was comparable or slightly higher than that observed under washed conditions (55–95% in Figure 8a). At 690 nm laser irradiation, however, the groups that were not washed exhibited higher photocytotoxicity (65–96% in Figure 8b) than those that were (55–95% in Figure 8a). This can be explained by the red-shifted Q band of PG-GW/Ce6 (Figure 5a), which allows more efficient excitation at 690 nm. The calculated *k*_PS_ and the higher photocytotoxicity under the conditions without washing support stronger singlet oxygen generation at this wavelength. Because 690 nm light penetrates deeper into tissues, this effect may be beneficial for in vivo PDT, as PG-GW/Ce6 located in the extracellular matrix in tumor tissue could still be activated to generate ROS. Considering the reported diffusion distance of singlet oxygen (100–150 nm) [44], this difference may be attributed to PG-GW/Ce6 adsorbed on the cell membrane in the groups that were not washed [45]. PG-GW/Ce6 can directly absorb 690 nm photons to generate singlet oxygen (Figure 7 and Figure S6), thereby contributing to the enhanced PDT.

Similar trends were observed in the case of 4T1 murine breast cancer cells, as shown in Figure 8c,d. Cell viability was 100% without light irradiation at Ce6 concentrations of up to 0.4 µg mL^−1^. Upon 660 nm light irradiation, viability dropped to 36–2% and 40–2% at a Ce6 concentration of ≥0.2 µg mL^−1^ with and without the washing step, respectively (Figure 8c). In contrast, the 690 nm laser irradiation groups that were and were not subjected to the washing process exhibited 83–59% and 74–35% viability, respectively (Figure 8d). The consistently higher survival of 4T1 cells compared to that for CT26 cells may be attributed to their faster proliferation rate, with a reported doubling time of ~12 h for 4T1 versus ~20 h for CT26 [46,47]. Nevertheless, both cell lines exhibited >98% photocytotoxicity at a Ce6 concentration of 0.4 μg mL^−1^ under 660 nm irradiation. Collectively, these findings suggest that under the tested conditions, irradiation at 660 nm is more effective than 690 nm for achieving robust PDT efficacy.

The minimum Ce6 concentration required to kill most of the cells via PG-GW/Ce6 under light irradiation is 3–38 times lower than the concentrations reported previously using Ce6 composites with GO [48,49,50], lanthanide-doped nanoparticles [51], and conjugated polymers [52], except with respect to graphene and MoS_2_ [23,24]. Although the experimental conditions regarding aspects such as laser power and cancer cell type were different, this comparison suggests that PG-GW delivers Ce6 into cells more efficiently than the other carriers.

## 4. Conclusions

PG-functionalized BM-GW Nfs (PG-GW) were developed as biocompatible and water-dispersible platforms. PG grafting endowed the BM-GW Nfs with high aqueous dispersibility, enabling their use in biomedical applications. The high X-ray absorption of BM-GW Nfs highlights the potential of PG-GW to serve as a multimodal sensitizer, and its improved dispersibility further enhances ROS generation under X-ray irradiation. Furthermore, PG-GW served as an effective carrier for Ce6, and the resulting PG-GW/Ce6 exhibited strong photocytotoxicity against cancer cells even at low Ce6 concentrations, confirming its efficacy in photodynamic therapy. Although these results demonstrate the promise of PG-GW/Ce6, this study is limited to in vitro evaluation and lacks in vivo pharmacokinetics data. Future work will focus on developing multimodal theranostics based on PG-GW/Ce6, integrating in vivo biodistribution studies, computed tomography imaging, and the combined therapeutic effects of Gd-NCT and PDT for translational cancer diagnosis and therapy.

## Data Availability

The original contributions presented in this study are included in the article and Supplementary Materials. Further inquiries can be directed to the corresponding author.

## Supplementary Materials

The following supporting information can be downloaded at: https://www.mdpi.com/article/10.3390/ma18225198/s1, Figure S1: (a) SEM image of GW-Nfs. Corresponding EDS elemental maps of (b) Gd, (c) W, and (d) O. (e) Map-summed EDS spectrum with atomic concentrations (%) extracted from the mapped area in (b–d).; Figure S2: (a) XRD patterns of GW Nfs (black) and BM-GW Nfs (red). (b) AFM image of BM-GW Nfs (scale bar: 410 nm).; Figure S3: Photographs of aqueous dispersions of BM-GW Nfs (left) and PG-GW (right) showing precipitation for BM-GW Nfs and stable dispersion for PG-GW. Figure S4: X-ray (150 kilovolt peak, kVp) sensitizability of BM-GW Nfs and PG-GW: ROS enhancement factor of BM-GW Nfs and PG-GW in relation to its BM-GW Nfs mass concentration in solution.; Figure S5: UV–vis absorption spectra (600–750 nm) of the ABDA assay solutions (100 μM) containing free Ce6 (black, red) or PG-GW/Ce6 (blue, green). Traces correspond to the irradiation conditions used in Figure 7: 660 nm (black, blue) and 690 nm (red, green).; Figure S6: Decomposition rate constants (*k*_PS_) of ABDA with free Ce6 under 690 nm and PG-GW/Ce6 under 660 nm or 690 nm irradiation. Each slope was determined by pseudo-first-order analysis of −ln(*A*_t_/*A*_0_) at 400 nm versus time.; Figure S7: Linear regression plot to determine IC50 for PDT with PG-GW/Ce6 after washing followed by 660 nm laser irradiation.; Figure S8: Live/dead images of CT26 cells obtained by trypan blue staining under different conditions: (a) control (no material and no irradiation), (b) 660 nm laser (1.3 W cm^−2^) irradiation only, (c) PG-GW/Ce6 (Ce6 concentration at 0.12 μg mL^−1^) without laser irradiation, and (d) PG-GW/Ce6 with 660 nm laser irradiation. All images were acquired at 10× magnification and scare bar: 200 μm.
