# Supplementary material for: Poly(glycerol)-Functionalized Gadolinium Tungstate Nanoflakes Loaded with Chlorin e6: Photodynamic Efficacy and Radiosensitization Potential for Multimodal Cancer Therapy
Source: Materials (Basel). 2025 Nov 16;18(22):5198. doi: 10.3390/ma18225198 (PMC12654580; doi:10.3390/ma18225198)
Supplement: Supplementary file 1 [file materials-18-05198-s001.zip › materials-3947588-supplementary.pdf]

Supporting Information

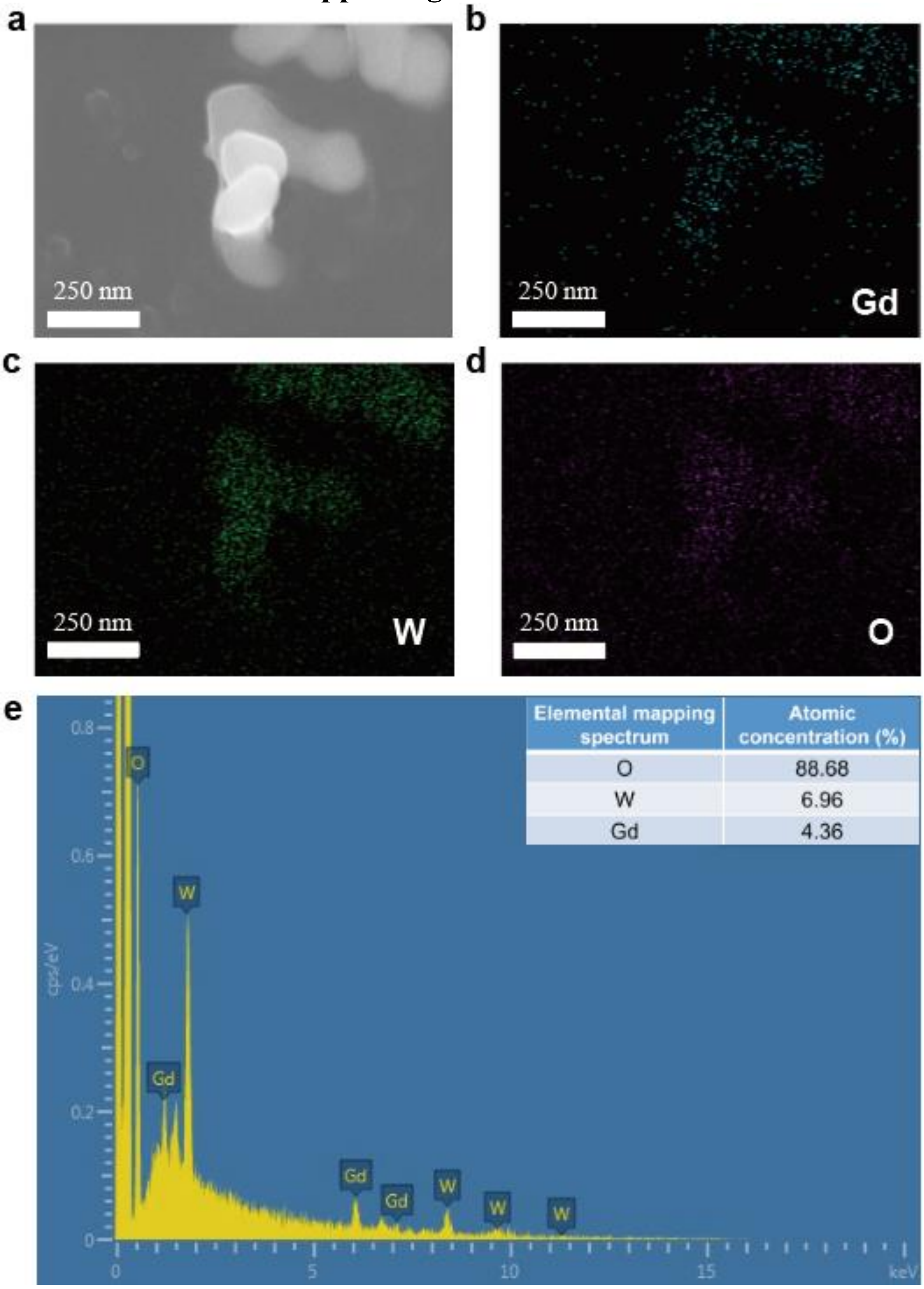

Figure S1. (a) SEM image of GW-Nfs. Corresponding EDS elemental maps of (b) Gd, (c) W, and (d) O. (e) Map-summed EDS spectrum with atomic concentrations (%) extracted from the mapped area in (b–d).

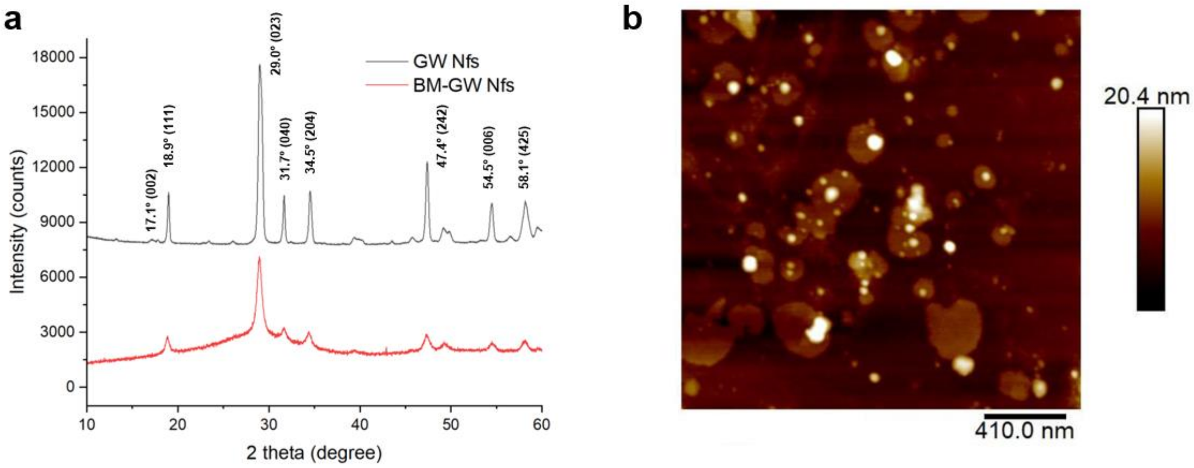

Figure S2. (a) XRD patterns of GW Nfs (black) and BM-GW Nfs (red). (b) AFM image of BM-GW Nfs (scale bar: 410 nm).

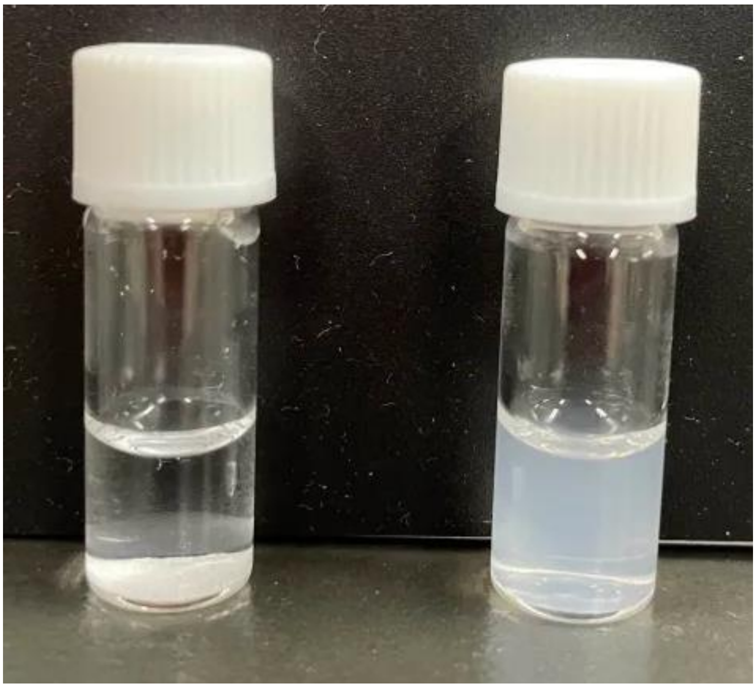

Figure S3. Photographs of aqueous dispersions of BM-GW Nfs (left) and PG-GW (right) showing precipitation for BM-GW Nfs and stable dispersion for PG-GW.

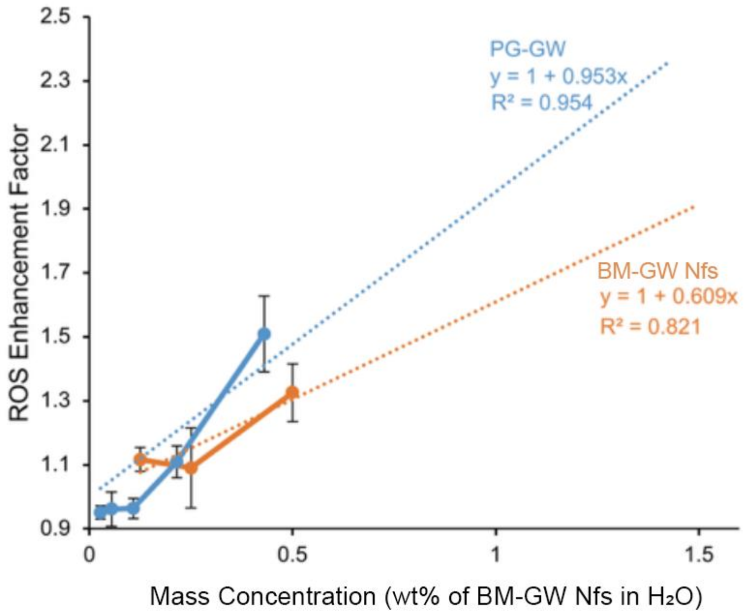

Figure S4. X-ray (150 kilovolt peak, kVp) sensitizability of BM-GW Nfs and PG-GW: ROS enhancement factor of BM-GW Nfs and PG-GW in relation to its BM-GW Nfs mass concentration in solution.

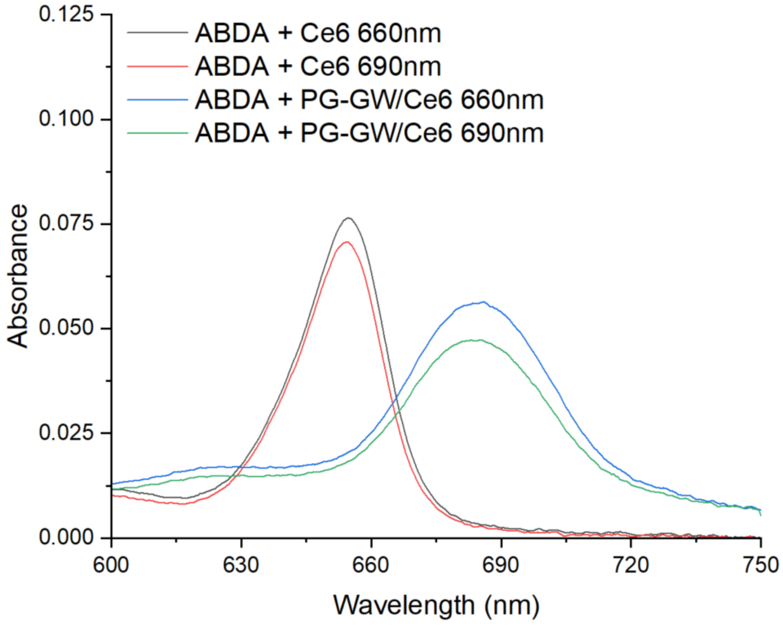

Figure S5. UV–vis absorption spectra (600–750 nm) of the ABDA assay solutions (100 μM) containing free Ce6 (black, red) or PG-GW/Ce6 (blue, green). Traces correspond to the irradiation conditions used in Figure 7: 660 nm (black, blue) and 690 nm (red, green).

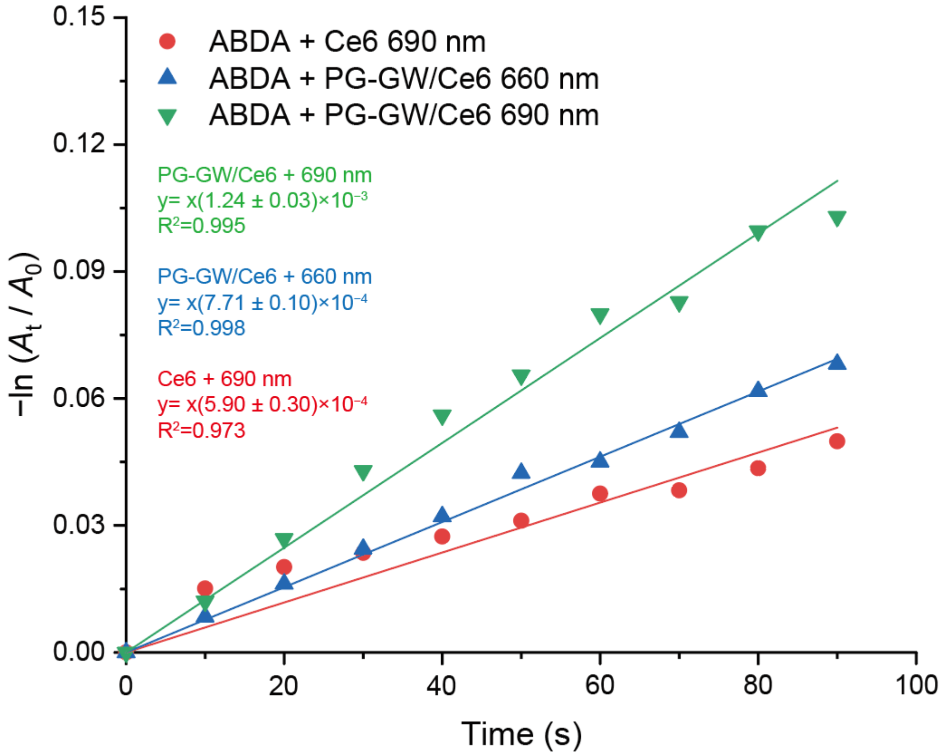

Figure S6. Decomposition rate constants (k_{PS}) of ABDA with free Ce6 under 690 nm and PG-GW/Ce6 under 660 nm or 690 nm irradiation. Each slope was determined by pseudo-first-order analysis of $-\ln(A_t/A_0)$ at 400 nm versus time.

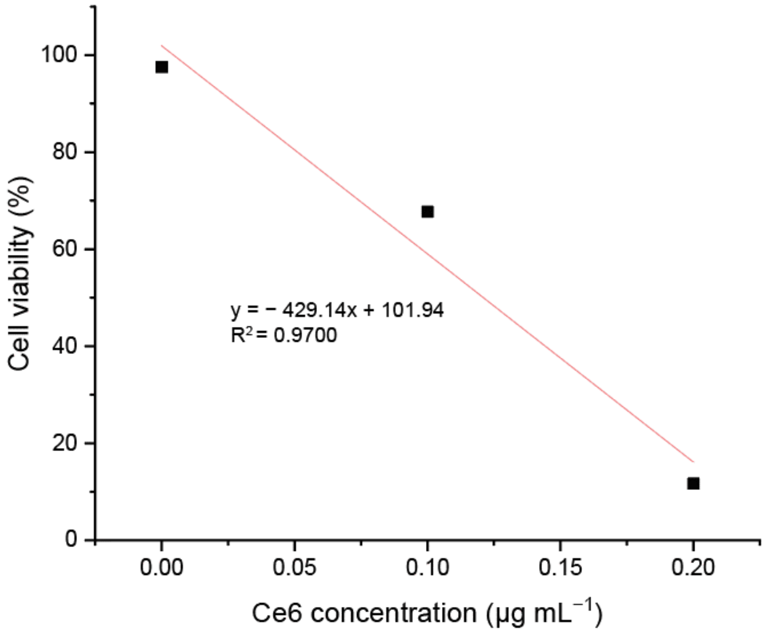

Figure S7. Linear regression plot to determine IC_{50} for PDT with PG-GW/Ce6 after washing followed by 660 nm laser irradiation.

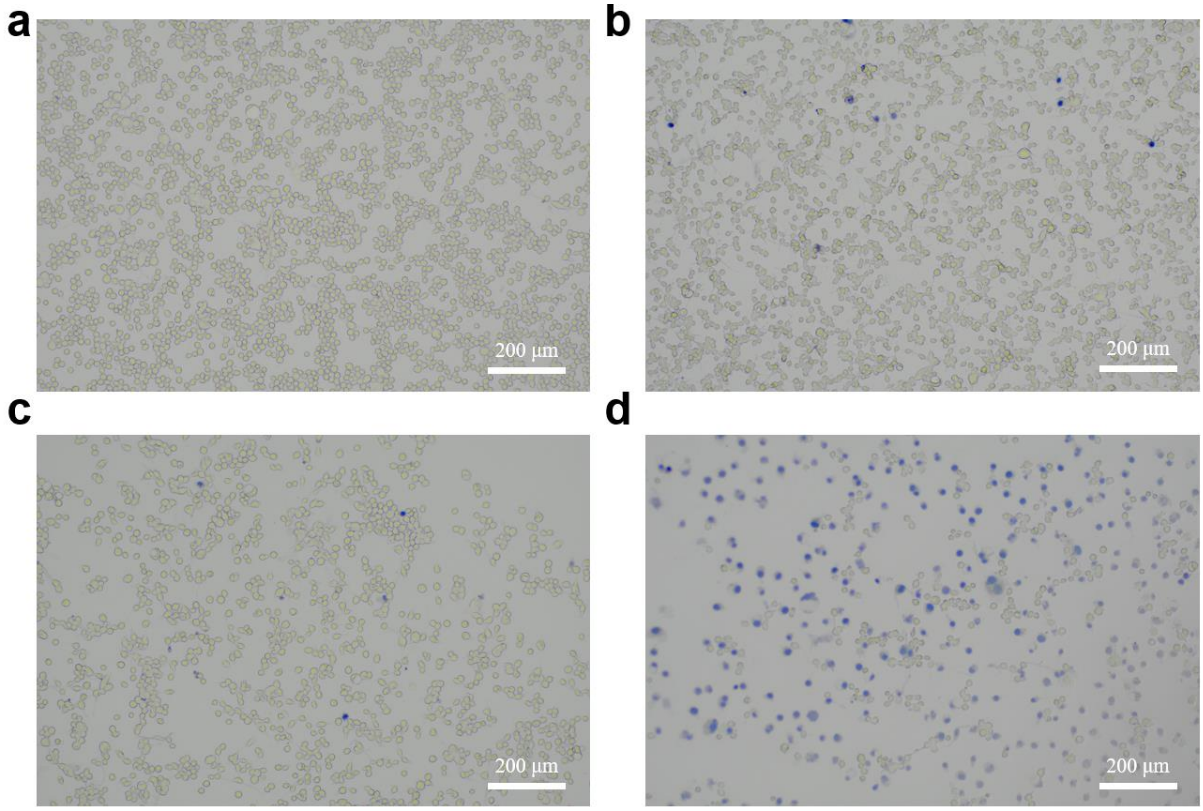

Figure S8. Live/dead images of CT26 cells obtained by trypan blue staining under different conditions: (a) control (no material and no irradiation), (b) 660 nm laser (1.3 W cm^{-2}) irradiation only, (c) PG-GW/Ce6 (Ce6 concentration at 0.12 µg mL^{-1}) without laser irradiation, and (d) PG-GW/Ce6 with 660 nm laser irradiation. All images were acquired at 10× magnification and scare bar: 200 µm.
